# Secondary Breast Cancer Sociodemographic Characteristics and Survival by Age Group

**DOI:** 10.1245/s10434-021-10340-3

**Published:** 2021-06-29

**Authors:** Candice A. M. Sauder, Qian Li, Richard J. Bold, Kathryn J. Ruddy, Theresa H. M. Keegan

**Affiliations:** 1 Division of Surgical Oncology, Department of Surgery, Davis Medical Center, University of California, Sacramento, CA USA; 2 Comprehensive Cancer Center, Davis Medical Center, University of California, Sacramento, CA USA; 3 Division of Hematology and Oncology, Center for Oncology Hematology Outcomes Research and Training (COHORT), University of California Davis School of Medicine, Sacramento, CA USA; 4 Department of Oncology, Mayo Clinic College of Medicine, Rochester, MN USA; 5 Division of Hematology and Oncology, Department of Internal Medicine, Davis Medical Center, University of California, Sacramento, CA USA

## Abstract

**Background:**

Secondary cancers account for 16% of all new cancer diagnoses, with breast cancer (BC) the most common secondary cancer. We have shown that secondary BC has unique characteristics and decreased survival compared with primary BC in adolescent and young adults (AYA; 15–39 years old). However, older BC populations are less well studied.

**Methods:**

Females (age ≥ 15 years) diagnosed with primary BC during 1991–2015 (n = 377,167) and enrolled in the California Cancer Registry were compared with those with secondary BC (n = 37,625) by age (15–39, 40–64, ≥ 65 years). We examined BC-specific survival (BCSS) accounting for other causes of death as a competing risk using multivariable Cox proportional hazards regression.

**Results:**

Most secondary BC patients were of older age (15–39, n = 777; 40–64, n = 15,848; ≥ 65, n = 21,000). Compared with primary BC treatment, secondary BCs were more often treated with mastectomy and less often with chemotherapy and/or radiation. BCSS was shorter in secondary BC patients than primary BC patients, but the survival difference between secondary and primary BC diminished with age [15–39 hazard ratio (HR): 2.09, 95% confidence interval (CI) 1.83–2.39; 40–64 HR: 1.51; 95% CI 1.44–1.58; ≥ 65 HR: 1.14; 95% CI 1.10–1.19]. Survival differences were most pronounced in women with hormone receptor positive disease and Hispanic and Asian/Pacific Islanders 40–64 years of age.

**Conclusions:**

When BC is diagnosed following a prior cancer of any organ site, BCSS is worse than when compared with patients for whom BC is the primary diagnosis, suggesting that we may need to tailor our treatments for women with secondary BC.

There are nearly 16.9 million cancer survivors living in the United States, and the number continues to rise with ongoing improvements in treatment and screening.^1^ In this extended lifetime of survivorship, many survivors of both childhood and adult-onset malignancies go on to develop a secondary malignancy, as more than 16% of new cancer diagnoses in the United States are second cancers.^2^ Breast cancer (BC) is the most common secondary malignancy in women over the age of 15, occurring after many types of primary malignancies, including a first primary BC.^3^ Multiple studies have shown that the adolescent and young adult (AYA) female population (15–39 years of age) has the highest absolute excess risk for secondary malignancies of any age group.^3^,^4^

For AYAs, primary and secondary BCs have distinct characteristics, with secondary BCs more often presenting with some good prognostic characteristics, including earlier stage, at low histologic grade, and with lymph node negativity.^5^-^7^ Despite this, being diagnosed with a secondary BC has been found to be an independent risk factor for worse overall survival in multivariable analysis. AYA survivors with a secondary BC had a 20% decreased relative survival at 5 years and an overall 1.58-fold increased risk of death compared with similar aged women with a primary BC.^5^,^6^ Survival and characteristics of secondary BCs in middle-aged (40–64 years old) and older women (65 years and older) are understudied to date, despite two thirds of all cancer survivors being 65 years or older.^8^

Therefore, we sought to better understand the clinical characteristics and survival of secondary breast malignancies by age. Using data from the large population-based California Cancer Registry (CCR), we examined demographics, clinical characteristics, and breast cancer-specific survival (BCSS) in AYA, middle-aged, and older women with secondary BC compared with primary BC. Findings from this study will help us to better understand how secondary cancers uniquely affect each of these age groups, potentially allowing tailored treatments for each population.

## Methods

### Patients

We obtained data from the CCR, which is one of the largest cancer registries in the world, participates in the National Cancer Institute’s Surveillance, Epidemiology, and End Results (SEER) program, and is estimated to include more than 99% of all invasive cancers diagnosed in California.^9^ We included in our analysis female California residents diagnosed with an invasive first and only BC or invasive BC as a second primary cancer after an invasive first primary cancer (soft tissue or hematologic malignancy) of any type (hereafter referred to as secondary BC) aged ≥ 15 during the period January 1, 1991, through December 31, 2015 [International Classification of Disease for Oncology, 3rd Edition, (ICD-O-3) site codes C50.0–50.9 (excluding codes for sarcoma, melanoma, neuroendocrine tumors, sweat gland tumors, and lymphoma for both primary and secondary BCs)]. Secondary BCs diagnosed within 2 months of the first primary cancer were excluded to remove what were likely multiple primary tumors. In addition, for women with two BCs, the secondary BC had to have a different histology from the first primary, occur in the contralateral breast, or occur > 5 years from the first primary.^5^,^10^

From the CCR database, we obtained information routinely recorded in the medical record at diagnosis for each primary and secondary BC patient on age at diagnosis, race/ethnicity (non-Hispanic White, Hispanic, non-Hispanic Black, and Asian or Pacific Islander, other/unknown), American Joint Committee on Cancer (AJCC) stage, tumor grade, histology, tumor size, lymph node involvement, estrogen receptor (ER), progesterone receptor (PR), and human epidermal growth factor receptor 2 (HER-2) tumor receptor expression status and sequence of primary cancer. The CCR has collected information on ER and PR since 1990 and on HER-2 since 1999.^11^,^12^ Because of HER-2 data incompleteness, we limited our analyses of HER-2 data to women diagnosed after 2003. ER/PR was defined as positive if either ER or PR was reported as positive, and triple-negative was defined as ER, PR, and HER-2 negative.

We also obtained registry information for each primary and secondary BC patient on initial course of treatment (surgery, chemotherapy, and radiation therapy), Census Block Group of residence at diagnosis, and vital status (routinely determined by the CCR through hospital follow-up and database linkages, including the Social Security Administration) as of December 31, 2015, and, for the deceased, the underlying cause of death. As information on patient education or other individual-level measures of socioeconomic status (SES) are not collected by the CCR, we assigned a previously developed measure of neighborhood SES based on patient address at time of diagnosis that incorporates Census Block Group on education, occupation, unemployment, household income, and poverty.^12^-^14^

**Fig. 1.**
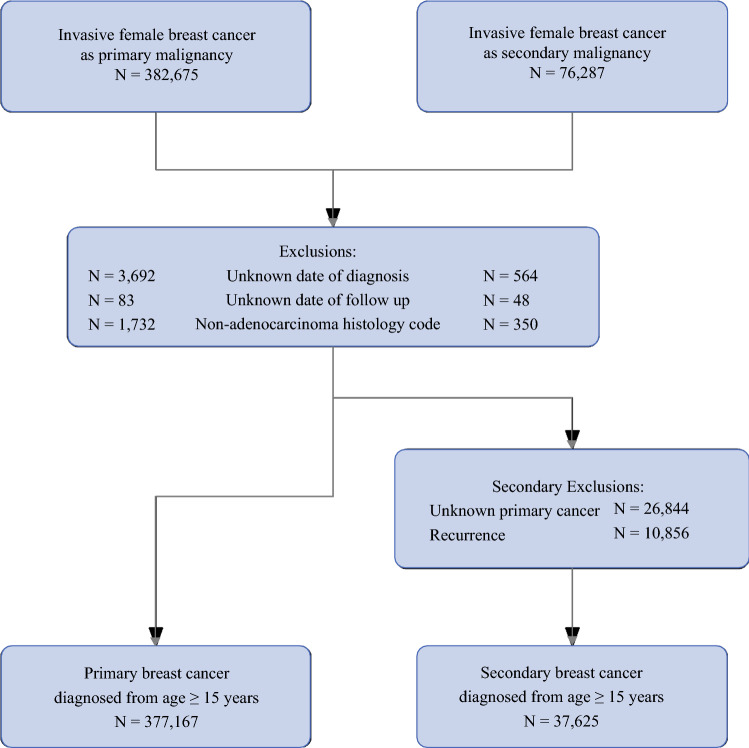
Selection of primary and secondary breast cancer cohorts from the California Cancer Registry (1991–2015). Exclusions are for the primary and secondary breast cancer diagnoses. Secondary exclusions are for women with secondary breast cancers

Of the 414,792 females diagnosed with a first and only, or secondary, invasive BC, we excluded patients with the histology codes noted above and unknown date of diagnosis or date of last follow-up. The resulting study population of females included 377,167 women with a first and only BC and 37,625 women with a secondary BC (Fig. 1). This study was approved by the University of California, Davis Institutional Review Board and by the California Committee for the Protection of Human Subjects.

### Statistical Analysis

Multivariable logistic regression was used to compare demographic (race/ethnicity, neighborhood socioeconomic status) and clinical (grade, histology, tumor size, lymph node involvement, ER, PR, and HER-2 status) factors between women with secondary versus primary BC by age group at BC diagnosis (15–39, 40–64, and ≥ 65 years). Results are presented as odds ratio (OR) and corresponding 95% confidence intervals (CI). Multivariable Cox proportional hazards regression models were used to evaluate associations of secondary versus primary BC, controlling for demographic and clinical factors, on breast cancer-specific survival (BCSS) by age group at BC diagnosis (15–39 years, 40–64 years, and ≥ 65 years). In addition, multivariable Cox proportional hazards regression models assessed the associations of secondary versus primary BC on BCSS by age group in subgroups defined by race/ethnicity and tumor receptor status. Effect modification was assessed between breast cancer (secondary vs primary) and each subgroup (age group and, within age group, by race/ethnicity and tumor receptors) by including interaction terms in the multivariable models. For deceased patients, survival time was measured from diagnosis date of the primary or secondary BC to the date of death from BC. Deaths from other causes were considered as competing risks. Patients alive at the study end date (31 December 2015) were censored at this date or at last known contact.

In all survival models, the proportional hazards assumption was assessed numerically based on cumulative sums of Martingale residuals and visually based on inspection of the survival curves [log (−log) of the survival distribution function by log (months)]; variables that violated this assumption were included as stratifying variables to allow for differing baseline hazards associated with these variables (chemotherapy, radiation, surgery, regional lymph nodes examined). Results are presented as hazard ratios (HR) and 95% confidence intervals (CI). Statistical analyses were performed using SAS statistical software (version 9.4), and a 2-sided *P* value of less than 0.05 was considered statistically significant.

## Results

A total of 37,625 females were diagnosed with an invasive BC as a secondary malignancy from 1991 to 2015 in the CCR. Of the cohort of secondary BC patients, most were of older age (15–39 years, n = 777; 40–64 years, n = 15,848; ≥ 65 years, n = 21,000). This was different from the age distribution for women diagnosed with primary BC (n = 377,167), where women aged 40–64 years comprised the largest proportion of patients (n = 205,101 vs 15–39 years, n = 23,298 and ≥ 65 years, n = 148,768) (Table 1).

**Table 1:** Characteristics and treatment of patients with primary and secondary breast cancer examined by age from the California Cancer Registry (1991–2015)

Characteristics	15–39 years at diagnosis		40–64 years at diagnosis		≥ 65 years at diagnosis	
	Only primary	Secondary	Only primary	Secondary	Only primary	Secondary
	N = 23,298	N = 777	N = 205,101	N = 15,848	N = 148,768	N = 21,000
	N (%)	N (%)	N (%)	N (%)	N (%)	N (%)
*Race/ethnicity*						
Non-Hispanic White	10,809 (46.4)	368 (47.4)	125,351 (61.1)	10,084 (63.6)	111,868 (75.2)	16,358 (77.9)
Non-Hispanic Black	1939 ( 8.3)	79 (10.2)	13,663 ( 6.7)	1284 ( 8.1)	7634 ( 5.1)	1190 ( 5.7)
Hispanic	6689 (28.7)	227 (29.2)	37,599 (18.3)	2599 (16.4)	16,424 (11.0)	1954 ( 9.3)
Asian/Pacific Islander	3581 (15.4)	101 (13.0)	25,830 (12.6)	1813 (11.4)	10,879 ( 7.3)	1403 ( 6.7)
Other/Unknown	280 ( 1.2)	<5 ( 0.3)	2658 ( 1.3)	68 ( 0.4)	1963 ( 1.3)	95 ( 0.5)
*Year of diagnosis*						
1991–1995	4362 (18.7)	75 ( 9.7)	28,016 (13.7)	633 ( 4.0)	26,672 (17.9)	846 ( 4.0)
1996–2000	4412 (18.9)	174 (22.4)	35,447 (17.3)	2052 (12.9)	28,822 (19.4)	2654 (12.6)
2001–2005	4603 (19.8)	197 (25.4)	42,456 (20.7)	3424 (21.6)	28,291 (19.0)	4130 (19.7)
2006–2010	4681 (20.1)	167 (21.5)	47,559 (23.2)	4532 (28.6)	29,802 (20.0)	5782 (27.5)
2011–2015	5240 (22.5)	164 (21.1)	51,623 (25.2)	5207 (32.9)	35,181 (23.6)	7588 (36.1)
*Neighborhood SES*						
Low SES	12,550 (53.9)	421 (54.2)	100,373 (48.9)	7546 (47.6)	77,370 (52.0)	10,101 (48.1)
High SES	10,748 (46.1)	356 (45.8)	104,728 (51.1)	8302 (52.4)	71,398 (48.0)	10,899 (51.9)
*AJCC Stage*						
Stage I	5843 (25.1)	279 (35.9)	86,774 (42.3)	8059 (50.9)	72,326 (48.6)	11,883 (56.6)
Stage II	11,186 (48.0)	264 (34.0)	77,877 (38.0)	4707 (29.7)	46,438 (31.2)	5766 (27.5)
Stage III	3605 (15.5)	104 (13.4)	21,184 (10.3)	1382 ( 8.7)	10,808 ( 7.3)	1419 ( 6.8)
Stage IV	1291 ( 5.5)	66 ( 8.5)	9355 ( 4.6)	815 ( 5.1)	7473 ( 5.0)	741 ( 3.5)
Unknown	1373 ( 5.9)	64 ( 8.2)	9911 ( 4.8)	885 ( 5.6)	11,723 ( 7.9)	1191 ( 5.7)
*Chemotherapy*						
Yes	17,183 (73.8)	454 (58.4)	102,042 (49.8)	6505 (41.0)	24,360 (16.4)	3127 (14.9)
No/Unknown	6115 (26.2)	323 (41.6)	103,059 (50.2)	9343 (59.0)	124,408 (83.6)	17,873 (85.1)
*Radiation*						
Yes	10,213 (43.8)	233 (30.0)	98,386 (48.0)	5216 (32.9)	57,478 (38.6)	6662 (31.7)
No/unknown	13,085 (56.2)	544 (70.0)	106,715 (52.0)	10,632 (67.1)	91,290 (61.4)	14,338 (68.3)
*Surgery*						
Lumpectomy	8538 (36.6)	230 (29.6)	105,056 (51.2)	6129 (38.7)	76,163 (51.2)	10,204 (48.6)
Mastectomy	12,786 (54.9)	456 (58.7)	85,862 (41.9)	8286 (52.3)	56,454 (37.9)	8900 (42.4)
None	1810 ( 7.8)	85 (10.9)	12,936 ( 6.3)	1318 ( 8.3)	13,786 ( 9.3)	1762 ( 8.4)
Unknown	164 ( 0.7)	6 ( 0.8)	1247 ( 0.6)	115 ( 0.7)	2365 ( 1.6)	134 ( 0.6)
*Tumor grade*						
Grade I	1578 ( 6.8)	55 ( 7.1)	37,070 (18.1)	3004 (19.0)	32,392 (21.8)	5231 (24.9)
Grade II	6829 (29.3)	223 (28.7)	76,486 (37.3)	6033 (38.1)	57,265 (38.5)	8883 (42.3)
Grade III	11,965 (51.4)	386 (49.7)	68,162 (33.2)	5069 (32.0)	34,861 (23.4)	4666 (22.2)
Undifferentiated	668 ( 2.9)	23 ( 3.0)	3445 ( 1.7)	242 ( 1.5)	1676 ( 1.1)	217 ( 1.0)
Unknown	2258 ( 9.7)	90 (11.6)	19,938 ( 9.7)	1500 ( 9.5)	22,574 (15.2)	2003 ( 9.5)
*Histology*						
Ductal	18,834 (80.8)	588 (75.7)	153,595 (74.9)	11,400 (71.9)	101,225 (68.0)	14,195 (67.6)
Lobular	2182 ( 9.4)	96 (12.4)	34,692 (16.9)	3048 (19.2)	28,553 (19.2)	4803 (22.9)
Other	2282 ( 9.8)	93 (12.0)	16,814 ( 8.2)	1400 ( 8.8)	18,990 (12.8)	2002 ( 9.5)
*Tumor size*						
T1a: ≤ 0.5 cm	878 ( 3.8)	68 ( 8.8)	14,215 ( 6.9)	1725 (10.9)	9379 ( 6.3)	2008 ( 9.6)
T1b: > 0.5–1 cm	1631 ( 7.0)	92 (11.8)	29,892 (14.6)	2973 (18.8)	26,471 (17.8)	4364 (20.8)
T1c: > 1–2 cm	6486 (27.8)	241 (31.0)	69,174 (33.7)	5232 (33.0)	50,791 (34.1)	7357 (35.0)
T2: > 2–5 cm	9638 (41.4)	210 (27.0)	63,719 (31.1)	3804 (24.0)	40,722 (27.4)	4985 (23.7)
T3: > 5 cm	2737 (11.7)	69 ( 8.9)	14,461 ( 7.1)	835 ( 5.3)	8387 ( 5.6)	909 ( 4.3)
Diffuse	469 ( 2.0)	16 ( 2.1)	2651 ( 1.3)	202 ( 1.3)	1377 ( 0.9)	136 ( 0.6)
Other	1459 ( 6.3)	81 (10.4)	10,989 ( 5.4)	1077 ( 6.8)	11,641 ( 7.8)	1241 ( 5.9)
*Lymph node involvement*						
Positive	11,491 (49.3)	294 (37.8)	75,584 (36.9)	4451 (28.1)	37,595 (25.3)	4343 (20.7)
Negative	10,844 (46.5)	416 (53.5)	122,525 (59.7)	10,715 (67.6)	98,857 (66.5)	15,621 (74.4)
Unknown	963 ( 4.1)	67 ( 8.6)	6992 ( 3.4)	682 ( 4.3)	12,316 ( 8.3)	1036 ( 4.9)
*Lymph nodes examined*						
Sentinel lymph node biopsy	6449 (27.7)	215 (27.7)	78,398 (38.2)	770 (42.7)	51,236 (34.4)	9009 (42.9)
Axillary lymph node dissection	14,299 (61.4)	375 (48.3)	106,850 (52.1)	6071 (38.3)	63,543 (42.7)	6559 (31.2)
No nodes examined/unknown	2550 (10.9)	187 (24.1)	19,853 ( 9.7)	3007 (19.0)	33,989 (22.8)	5432 (25.9)
*Estrogen receptor status*						
Positive	12,989 (55.8)	366 (47.1)	140,906 (68.7)	10,720 (67.6)	103,416 (69.5)	15,772 (75.1)
Negative	7215 (31.0)	279 (35.9)	40,267 (19.6)	3361 (21.2)	18,848 (12.7)	2820 (13.4)
Unknown	3094 (13.3)	132 (17.0)	23,928 (11.7)	1767 (11.1)	26,504 (17.8)	2408 (11.5)
*Progesterone receptor status*						
Positive	11,454 (49.2)	330 (42.5)	119,812 (58.4)	8592 (54.2)	84,423 (56.7)	12,675 (60.4)
Negative	8482 (36.4)	304 (39.1)	57,441 (28.0)	5196 (32.8)	34,987 (23.5)	5565 (26.5)
Unknown	3362 (14.4)	143 (18.4)	27,848 (13.6)	2060 (13.0)	29,358 (19.7)	2760 (13.1)
*HER-2 status*^a^						
Positive	3366 (26.5)	104 (23.2)	24,523 (19.6)	2069 (17.4)	10,983 (13.5)	2014 (12.6)
Negative	8222 (64.7)	277 (61.7)	87,563 (70.1)	8402 (70.5)	59,081 (72.6)	11,904 (74.7)
Unknown	1113 ( 8.8)	68 (15.1)	12,829 (10.3)	1454 (12.2)	11,326 (13.9)	2022 (12.7)

SES = Socioeconomic status; AJCC = American Joint Committee on Cancer; HER-2 = Human epidermal growth factor receptor-2

^a^HER-2 data is limited to 2003+ diagnoses

In the secondary BC cohort, women ≥ 65 years were less likely to be non-Hispanic (NH) Black (5.7% vs 15–39 years, 10.2% and 40–64 years, 8.1%), Hispanic (9.3% vs 15–39 years, 29.2% and 40–64 years, 16.4%), or Asian/Pacific Islander (6.7% vs 15–39 years, 13.0% and 40–64 years, 11.4%) race/ethnicity than younger women. Across all ages, women with secondary BC had smaller (T3: 15–39 years, 8.9% vs11.7%; 40–64 years, 5.3% vs 7.1%; ≥ 65 years, 4.3% vs 5.6%) and more lymph node negative (15–39 years, 37.8% vs 49.3%; 40–64 years, 28.1% vs 36.9%; ≥ 65 years, 20.7% vs 25.3%) tumors than their primary BC counterparts. Additionally, the percentage of secondary BC tumors that were ER and PR positive and HER-2 negative increased with age. However, regardless of age, secondary BC patients were more likely to be treated with mastectomy and less likely to receive chemotherapy or radiation.

In the multivariable logistic regression models, secondary BCs were more likely to occur among non-Hispanic Black AYAs (OR: 1.25; CI 0.97–1.61; borderline statistical significance) and middle-aged women (OR: 1.14; CI 1.07–1.21), but less likely to occur among middle-aged (OR: 0.80; CI 0.76–0.84) and older (OR: 0.72; CI 0.68–0.75) Hispanics and Asian/Pacific Islanders compared with non-Hispanic Whites in each age group (Table 2). Secondary BCs were more likely to be of higher grade at all ages, but this association did not reach statistical significance for women aged 15–39 years at diagnosis. In all age groups, secondary BCs were more likely to have lobular histology, be smaller in size, be lymph node negative and be ER/PR negative than primary BCs. HER-2 status was similar for women with primary and secondary BC by age.

**Table 2:** Adjusted^a^ logistic regression model of factors associated with having secondary breast cancer compared with primary breast cancer

Characteristics	15–39 years at diagnosis	40–64 years at diagnosis	≥ 65 years at diagnosis
	OR (95% CI)	OR (95% CI)	OR (95% CI)
*Race/ethnicity*			
Non-Hispanic White	Reference	Reference	Reference
Non-Hispanic Black	1.25 (0.97,1.61)	1.14 (1.07,1.21)	1.01 (0.95,1.08)
Hispanic	1.02 (0.86,1.21)	0.80 (0.76,0.84)	0.72 (0.68,0.75)
Asian/Pacific Islander	0.80 (0.64,1.01)	0.80 (0.76,0.84)	0.73 (0.69,0.77)
*Tumor grade*			
Grade I	Reference	Reference	Reference
Grade II	1.17 (0.86,1.60)	1.14 (1.09,1.20)	1.08 (1.04,1.12)
Grade III	1.25 (0.92,1.71)	1.18 (1.12,1.25)	1.06 (1.01,1.11)
Undifferentiated	1.18 (0.71,1.97)	1.13 (0.98,1.30)	1.16 (1.00,1.35)
*Histology*			
Ductal	Reference	Reference	Reference
Lobular	1.53 (1.22,1.92)	1.24 (1.19,1.30)	1.20 (1.16,1.25)
*Tumor size*			
T1a: ≤ 0.5 cm	Reference	Reference	Reference
T1b: > 0.5–1 cm	0.80 (0.58,1.12)	0.90 (0.84,0.96)	0.82 (0.77,0.87)
T1c: > 1–2 cm	0.52 (0.39,0.69)	0.69 (0.65,0.73)	0.72 (0.68,0.76)
T2: > 2–5 cm	0.29 (0.22,0.39)	0.55 (0.51,0.58)	0.62 (0.58,0.66)
T3: > 5 cm	0.33 (0.23,0.47)	0.52 (0.48,0.57)	0.54 (0.50,0.60)
Diffuse	0.37 (0.20,0.66)	0.81 (0.69,0.95)	0.78 (0.64,0.94)
*Lymph node involvement*			
Negative	Reference	Reference	Reference
Positive	0.80 (0.68,0.94)	0.77 (0.74,0.80)	0.82 (0.79,0.85)
*ER and PR status*			
Positive	Reference	Reference	Reference
Negative	1.39 (1.17,1.67)	1.26 (1.21,1.32)	1.11 (1.06,1.17)
*HER-2 status*^b^			
Positive	Reference	Reference	Reference
Negative	1.09 (0.87,1.38)	1.12 (1.06,1.18)	1.03 (0.97,1.08)

The estimates of odds ratios for unknown groups are not present.

OR = Odds ratio; CI = Confidence interval; ER = Estrogen receptor; PR = Progesterone receptor; HER-2 = Human epidermal growth factor receptor-2.

^a^Models adjusted for all variables in the Table as well as year of diagnosis and socioeconomic status.

^b^HER-2 data is limited to 2003+ diagnoses.

**Fig. 2.**
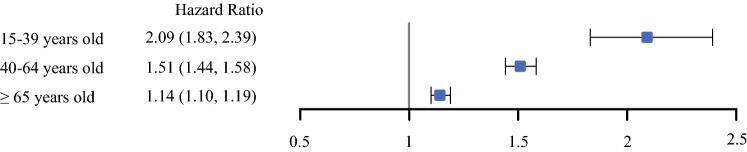
Adjusted breast cancer-specific survival associated with having secondary breast cancer compared with primary breast cancer by age group. Each age group was evaluated in a separate model. Models were adjusted for race/ethnicity, year of diagnosis, neighborhood socioeconomic status, tumor size, lymph node involvement, grade, tumor receptors, and histology; and stratified by chemotherapy, radiation, surgery, and regional nodes examined

**Fig. 3.**
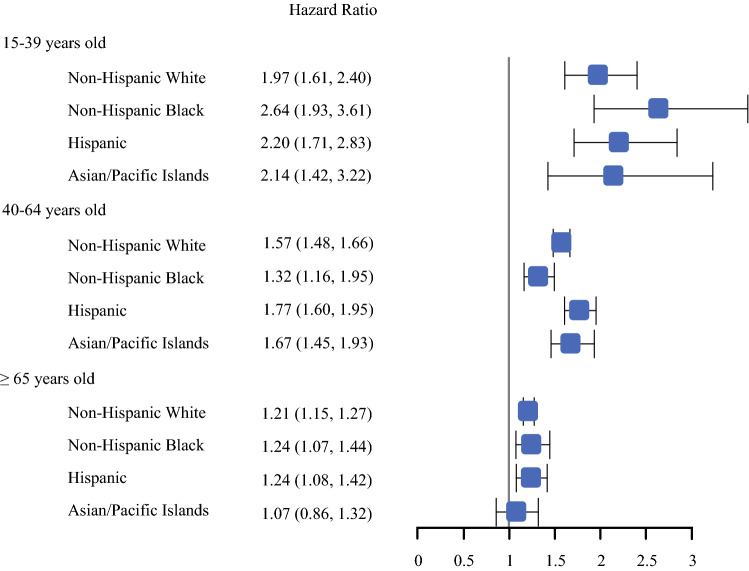
Adjusted breast cancer-specific survival associated with having secondary breast cancer compared with primary breast cancer by age group and race/ethnicity. Each age and race/ethnicity group was evaluated in a separate model. Models were adjusted for year of diagnosis, neighborhood socioeconomic status, tumor size, lymph node involvement, grade, tumor receptors, and histology; and stratified by chemotherapy, radiation, surgery, and regional nodes examined

In multivariable survival models evaluating the impact of secondary versus primary BC, we found that the hazard of breast cancer death was higher for women with secondary BCs in all age groups, but the impact on survival diminished with age (15-39 years old, HR: 2.09, CI 1.83–2.39; 40–64 years old, HR: 1.51; CI 1.44–1.58, ≥ 65 years old, HR: 1.14; CI 1.10–1.19) (*p* for interaction < 0.001) (Fig. 2). Within age groups, the negative impact of secondary (vs primary) BC on BCSS persisted in all racial/ethnic groups, except Asian/Pacific Islanders ≥ 65 years (Fig. 3). While the negative impact of secondary BC did not differ significantly by race/ethnicity in AYAs (*p* = 0.30) or women ≥ 65 years (*p* = 0.59), the survival difference of a secondary BC compared with primary BC appeared to be greatest among non-Hispanic Black AYAs and least among Asian/Pacific Islanders ≥ 65 years. Racial/ethnic differences in survival were observed in middle-aged women (*p* = 0.03), with the negative impact of secondary BC more pronounced for Hispanics and Asian/Pacific Islanders than non-Hispanic Blacks.

**Fig. 4.**
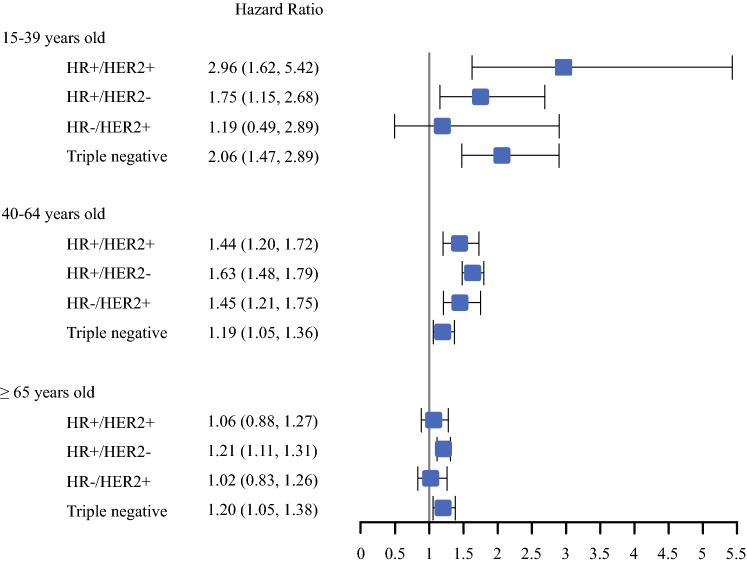
Adjusted breast cancer-specific survival associated with having secondary breast cancer compared with primary breast cancer by age group and tumor receptors. Each age and tumor marker group was evaluated in a separate model. Models were adjusted for race/ethnicity, year of diagnosis, neighborhood socioeconomic status, tumor size, lymph node involvement, grade, and histology; and stratified by chemotherapy, radiation, surgery, and regional nodes examined. HR = hormone receptor

In a subset of women diagnosed after 2003, we found that BCSS differences between secondary and primary BC based on tumor receptor status were evident in women 40–64 years and ≥ 65 years old (*p* < 0.001) (with borderline differences observed in AYAs based on tumor receptor status; *p* = 0.07) (Fig. 4). The impact of BCSS for secondary compared with primary BC was most pronounced amongst women with hormone receptor positive, HER-2 negative disease across all ages. For women with HER-2 positive tumors, regardless of hormone receptor status, BCSS was only worse for secondary BCs amongst women 40–64 years old. BCSS was also significantly decreased in triple negative secondary BCs at all ages, but to a lesser extent.

## Discussion

In this large, population-based study, we found that BCSS was worse for women with secondary than primary BCs, and this difference was largest in the 15–39 age group, consistent with prior work.^2^,^4^,^7^ We additionally identified that the impact of secondary BC on survival differs by race/ethnicity and tumor subtype. Among 40- to 64-year-old women, the negative impact of secondary BC on BCSS was more pronounced among Hispanics and Asian/Pacific Islanders than non-Hispanic Blacks. However, this survival difference between secondary and primary BC was no longer seen in Asian/Pacific Islanders ≥ 65 years. Additionally, the negative impact of secondary BC was particularly substantial in women with hormone receptor positive BCs. This is the first study, to our knowledge, that examines secondary BC characteristics and outcomes compared with primary BCs across the age spectrum.

Little is known about survival in older patients with secondary BC, which is surprising as the majority of cancer survivors are 65 years of age or older.^8^ In primary BC, the best survival rates have been found for middle-aged women, with decreased survival at each end of the age spectrum.^15^–^17^ Confirming prior findings, we identified that AYA women with secondary BC experienced significantly worse BCSS than AYAs with primary BC.^6^,^7^ This may be due to the inherent aggressive nature of these tumors, which can arise in radiated tissue after mantle or chest radiation, and may be genetically less treatment-responsive, such as triple negative tumors which are more likely found in AYA *BRCA* mutation carriers. Additionally, we found the most minimal BCSS difference between secondary and primary BCs in older women, perhaps because they tend to develop small, low-grade, hormone receptor positive tumors in both the primary and secondary cancer settings. It is well known that these types of BCs have relatively good prognosis in the primary BC setting, and our findings suggest that they have a similar prognosis in the secondary BC setting in older women as well.

Many studies have demonstrated differences in BC incidence rates and survival by race/ethnicity,^18^–^22^ but few studies have considered the impact of secondary BC (vs primary BC) by race/ethnicity and age. In our study, non-Hispanic Black women are more likely to have a second BC at all ages, but the difference in BCSS between secondary and primary BCs is less in middle-aged non-Hispanic Blacks than in all other racial/ethnic groups. This is most likely due to non-Hispanic Black women having poorer survival after primary BC compared with women of other race/ethnicities,^23^,^24^ thus making the difference in BCSS between the primary and secondary BCs less impactful. In contrast, Asian/Pacific Islanders and Hispanics have been found to have better primary BC prognosis than non-Hispanic Whites,^25^–^27^ which may have contributed to the significantly more pronounced detrimental impact of secondary BC on survival in middle-aged Asian/Pacific Islander and Hispanic women observed in our study. In women over 65 years, there was no difference in survival for Asian/Pacific Islanders between primary vs secondary BC. Our data show that there is something innately different about secondary BCs that modifies the differences seen traditionally by race/ethnicity, especially for middle-aged women who traditionally have the best survival. This may be related to types of cancer-predisposing treatment received previously (e.g., radiation) and genetic risk factors that are more common in certain racial and ethnic groups predisposing to specific types of malignancies. Additionally, the decreased BCSS may be related to prior treatment regimens received for their primary cancer impacting their further use for secondary BC treatment, such as mantle radiation being used to treat lymphoma, thus eliminating it from the armamentarium available for a patient’s secondary BC treatment.

Breast cancer is a complex and heterogeneous disease for which therapeutic molecular markers are known to affect BCSS. In primary BC, hormone (estrogen/progesterone) receptor positive BC has been shown to have the best BCSS, and many women with these tumor types get de-escalated care at older ages for this subtype of disease.^28^ Not much is known about molecular markers and survival in secondary BC. In middle-aged and older women, the impact of secondary BC on BCSS was most pronounced among women who have hormone receptor positive disease (ER/PR positive, HER-2 negative), the most common tumor subtype in this age group.^7^,^29^ We also observed that middle-aged women with HER-2 positive or triple negative tumors had fewer differences in BCSS between primary and secondary BC than hormone receptor positive disease, which might stem from the already decreased BCSS seen in these subtypes in primary BC compared with hormone receptor positive BCs.

Our database has limitations in that we lacked full treatment details. The radiation fields are not specified, and chemotherapeutic agents are unknown. In addition, there are no genetic data collected, which can influence treatment decisions and risk for secondary BC, especially for patients who have a *BRCA* mutation. Additionally, due to the nature of the database, there is no information about treatment failure (e.g., locoregional or distant recurrences). Lastly, the smaller secondary BC cohort in AYAs (2% of secondary BC in our study) could have impacted our ability to identify differences in patient subgroups and should be the focus of future research. Despite these limitations, the current study represents over 99% of all cancers diagnosed in California and includes a racially and ethnically diverse set of patients from a population-based registry, increasing the generalizability of our results.

In conclusion, we found that BCSS is significantly decreased among all women that develop a secondary BC compared with those with a primary BC; however, BCSS differences between secondary and primary BC are greatest for women who have hormone receptor positive disease or are of Hispanic and Asian/Pacific Islander ethnicity: groups of women who often have superior survival after primary BC. Additional research is needed to help translate these findings into improved individualized treatments and screenings for secondary BC amongst women with poor prognosis.
